# The bacterial association with oral cavity and intra-abdominal abscess after gastrectomy

**DOI:** 10.1371/journal.pone.0242091

**Published:** 2020-11-09

**Authors:** Mao Nishikawa, Michitaka Honda, Ryosuke Kimura, Ayaka Kobayashi, Yuji Yamaguchi, Soshi Hori, Hiroshi Kobayashi, Mitsuru Waragai, Hidetaka Kawamura, Yujiro Nakayama, Yukitoshi Todate, Yoshinao Takano, Hisashi Yamaguchi, Koichi Hamada, Susumu Iketani, Ichiro Seto, Yuichi Izumi, Kanichi Seto

**Affiliations:** 1 Department of Oral and Maxillofacial Surgery, Southern TOHOKU General Hospital, Koriyama, Fukushima, Japan; 2 Department of Minimally Invasive Surgical and Medical Oncology, Fukushima Medical University, Fukushima, Fukushima, Japan; 3 Department of Surgery, Southern TOHOKU General Hospital, Koriyama, Fukushima, Japan; Loma Linda University Health School of Medicine, UNITED STATES

## Abstract

**Background:**

Perioperative oral management has been reported to be effective for preventing postoperative infectious complications. In addition, severe periodontal disease was identified as the significant risk factor for complications after gastrointestinal surgery. We investigated the bacteriological association between the periodontal pocket, stomach mucosa and drainage fluid to determine whether oral bacteria directly cause intra-abdominal infection after gastrectomy.

**Methods:**

Patients who were scheduled to undergo surgery for gastric cancer were prospectively enrolled. We evaluated the similarity of bacterial strains in periodontal pocket, stomach mucosa and fluid from drainage tube. Gingival crevicular fluid and dental plaque were collected from the periodontal pocket and cultured to detect bacteria. Specimens from the resected stomach were collected and used for bacterial culturing. Drainage fluid from the abdominal cavity was also cultured.

**Results:**

All of 52 patients were enrolled. In the periodontal pocket, *α-Streptococcus* spp., *Neisseria* sp., and *Prevotella* sp. were mainly detected. Bacterial cultures in the stomach mucosa were positive in 26 cases. In 20 cases (76.9%), the detected strains were the same as those in the periodontal pocket. Six patients had the postoperative intra-abdominal infection after gastrectomy, and the same bacterial strains was detected in both of drainage fluid and periodontal pocket in two patients with severe periodontal disease.

**Conclusions:**

We found the bacteriological association that same strain detected in periodontal pocket, stomach and in intra-abdominal drainage fluid after gastrectomy in patients with periodontal disease.

## Introduction

Perioperative oral management has been reported to be effective for preventing postoperative infectious complications (POICs) such as aspiration pneumonia after esophagectomy or other gastrointestinal surgeries [1–4]. We previously reported that the severity of periodontal disease (PD), as determined by the probing pocket depth, is an important risk factor for POICs after gastrointestinal surgery [5]. While it is easy to understand how the surgical wound or respiratory tract is directly exposed to bacteria from the oral cavity in esophagectomy or head and neck surgery, the infectious route has been still unclear in the other abdominal surgery.

It is considered that chronic inflammation in the periodontal pockets might cause bacteremia [6–8], or that patients with severe PD might have other lifestyle-related diseases related to POICs [7, 9–12]. However, the possibility that the bacteria in the periodontal pocket directly cause intra-abdominal infection in cases of severe PD as an infectious route should also be considered. In gastrectomy in particular, large numbers of bacteria may enter the stomach via the oral cavity and leak into the abdominal cavity during anastomosis. If the bacteria in the periodontal pocket could also be causative pathogen of intra-abdominal abscess after gastrectomy, the treatment of PD would be one of the most important thing among preoperative managements.

We conducted this study to investigate two clinical questions: 1) Are oral bacteria in patients with PD ingested and present in the stomach; and 2) Can bacteria in the stomach be a causative pathogen of intra-abdominal infection after gastrectomy.

## Methods

### Patients enrollment

This study protocol was approved by the Institutional Review Board (UMIN000033851) and was conducted in accordance with the Declaration of Helsinki and national ethical regulations. Informed consent was obtained from all participating patients.

This was a single-institute and prospective cohort study. Patients for whom radical resection was planned for gastric cancer from November 2017 to July 2019 were considered for enrollment in this study. The inclusion criterion was planned radical resection for gastric adenocarcinoma. Cases involving emergency surgery, edentulous patients or patients with only tooth stumps, patients who were taking antibiotics before surgery and patients with other infectious conditions were excluded.

### Oral management and evaluation of PD

**Fig 1:**
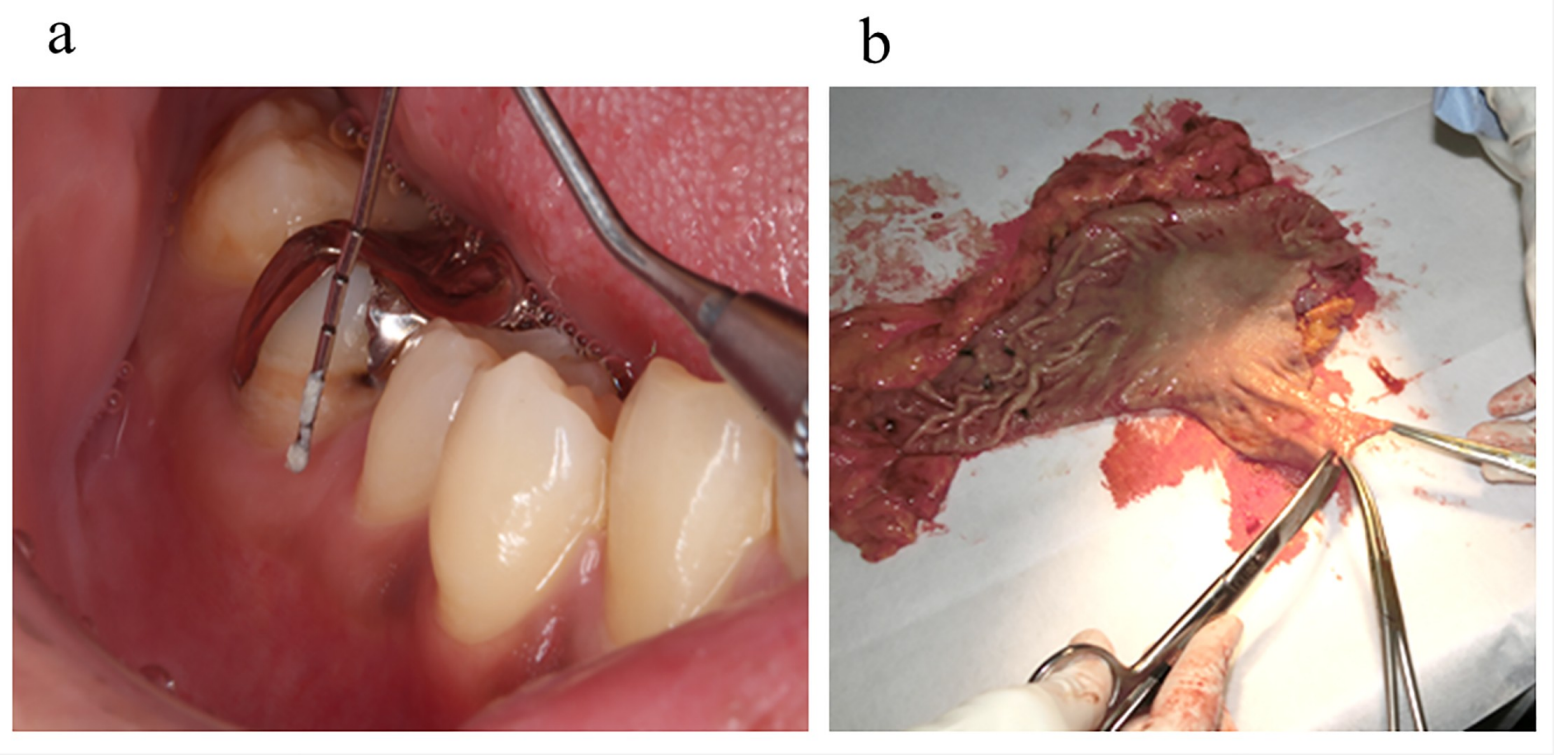
Sample collection. Samples were collected from the periodontal pocket and stomach mucosa. a: Gingival crevicular fluid and dental plaque were collected from the periodontal pocket and cultured. b: The stomach specimen was cut open and a section of the stomach mucosa (20x20mm) was collected under sterile conditions.

In all patients, we obtained informed consent for written and the oral cavity was evaluated by a dentist and oral management was performed. We previously reported the details [5]. In brief, the dentist examined patients’ oral cavity and the severity of PD. Orthopantomography was performed in all patients. The dentists and dental hygienists performed scaling and oral cleaning as well as tooth brushing instruction. The dentist also described the patients’ oral condition and the pocket probing depth (PPD). PD was classified into 3 categories in accordance with the Periodontal Treatment Guidelines 2015 as follows: mild (the deepest PPD < 4 mm), intermediate (the deepest PPD 4 mm to < 6 mm), or severe ((the deepest PPD ≥ 6 mm) [13]. Gingival crevicular fluid and dental plaque were collected from the periodontal pocket with a pocket probe and cultured for bacteria (Fig 1).

### Surgical procedure and postoperative management

All patients were treated with prophylactic antibiotics (cefazolin sodium [1.0 g]) at least 30 minutes before skin incision and during the operation every 3 h. The surgeon performed gastrectomy with lymph node dissection in accordance with the gastric cancer treatment guidelines [14]. The use of a laparoscope or robot, and method of reconstruction were selected by the surgeon. After gastric resection, the stomach specimen was cut open, and a section of the stomach mucosa (20 × 20 mm) was collected under sterile conditions (Fig 1). In all cases, a 19-Fr suction drain was placed in the supra-pancreatic space. Postoperative management was performed according to the clinical pathway in the hospital. Culturing of drainage fluid from the intra-abdominal drain tube was performed. Antibiotics were not routinely administered to patients without physical signs of infectious complications.

### Bacterial culture

The specimens of gingival exudate or plaque in the periodontal pocket, stomach mucosa and drainage fluid from the abdominal cavity were stored in an anaerobic culture tube II (TERUMO Corporation, Tokyo Japan). A smear test, aerobic culturing and anaerobic culturing were performed using TSA II with 5% sheep’s blood/chocolate II medium (Becton, Dickinson and Co., NJ, USA), Bromothymol Blue Lactose medium (Becton, Dickinson and Co., NJ, USA), Anaero Columbia RS blood medium (Becton, Dickinson and Co., NJ, USA) and CO_2_ incubation. Pure culture and identification were performed the day after culturing. Aerobic bacteria were identified using a MicroScan WalkAway 96 Plus (Becton, Dickinson and Co., NJ, USA), and anaerobic bacteria were identified using a RapID ANA II (Thermo Fisher Scientific, MA, USA).

### Outcomes and statistics

The aim of this study was to confirmed whether or not the bacteria in the periodontal pockets could cause intra-abdominal abscess after gastrectomy. To reject the null hypothesis that these bacteria were not associated with abscess formation at all, we decided to evaluate at least five patients who had intra-abdominal infection after gastrectomy. Assuming that the incidence of grade 2 or more severe intra-abdominal infection, including organ space surgical site infection (SSI), anastomotic leakage or intra-abdominal abscess was 10%, we estimated that at least 50 cases were needed to investigate the bacteriological relationship between oral bacteria and intra-abdominal infection. If we found any concordance between the bacteria in the periodontal pockets, stomach mucosa and drainage fluid from the abdominal cavity, the null hypothesis would be rejected. We also evaluated the descriptive statistics of bacterial strains in the periodontal pocket, stomach mucosa and drainage fluid according to PD severity.

All statistical analyses were performed with the STATA software program, version 14.

## Results

All 52 patients who underwent gastric cancer surgery during November 2017 to April 2019 were enrolled. Table 1 shows the patients’ characteristics. All POICs were evident in 12 patients (23.1%), including intra-abdominal fluid collection or abscess formation (n = 6), superficial SSI (n = 3), anastomotic leakage (n = 1), pneumonia (n = 1) and urinary tract infection (n = 1). Grade ≥2 intra-abdominal infection occurred in 7 cases (13.5%). Among these cases, the cultures of drainage fluid were positive in 6 (11.5%).

**Table 1:** Patients’ characteristics.

		Number of Patients (n = 52)	%
Age	median [IQR]	66.5 [59–71.5]	
	<75	43	82.7
	≥75	9	17.3
Sex	Male	35	67.3
	Female	17	32.7
BMI (kg/m^2^)	mean (SD)	23.1 (3.31)	
Performance	0	49	94.2
Status	1	2	3.8
(ECOG)	2	1	1.9
Smoking	Non / Mild (<5PY)	24	46.2
	Intermediate (≥5, <50PY)	18	34.6
	Heavy (≥50PY)	8	15.4
Comorbidities	Diabetes	7	13.5
	Hypertension	22	42.3
	COPD	2	3.8
	Hemodialysis	1	1.9
Procedure	TG	10	19.2
	DG	39	75.0
	PG	3	5.8
Approach	Open surgery	9	17.3
	Laparoscopic surgery	38	73.1
	Robotic surgery.	5	9.6
Use of PPI before surgery		35	67.3
Periodontal	Mild	5	9.6
disease	Intermediate	22	42.3
	Severe	25	48.1
TNM classification	T1/T2/T3/T4	34/7/2/9	65.4/13.5/3.8/17.3
	N0/N1/N2/N3	42/3/6/1	80.8/5.8/11.5/1.9
	M0/M1	49/0	94.2/5.8

IQR, Inter quadrant range; BMI, Body mass index, SD, Standard deviation, PY: Pack-years; COPD, Chronic obstructive pulmonary disease; TG, Total gastrectomy; DG, Distal gastrectomy; PG, Proximal gastrectomy; PPI, Proton pump inhibitor.

**Fig 2:**
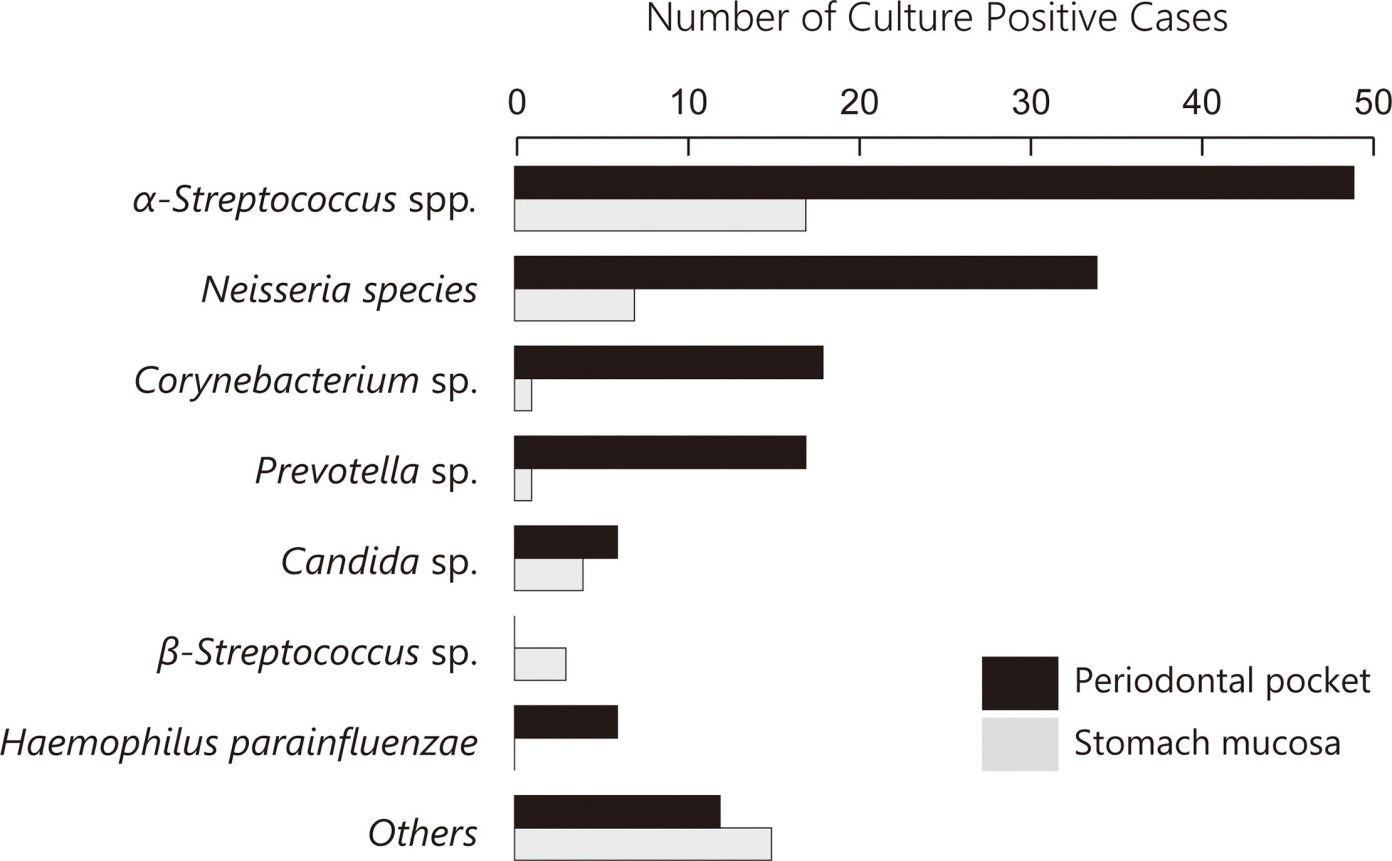
The bacterial strains in the periodontal pocket and stomach mucosa. The black and gray bars show the positive number of each strain in periodontal pocket and stomach mucosa, respectively.

Fig 2 shows the bacterial strains detected in the periodontal pocket, stomach mucosa and drainage fluid. Periodontal pocket cultures were positive in all cases. *α-Streptococcus* spp., *Neisseria* sp. were common in the oral cavity. *Prevotella* sp., which was closely related to deterioration of PD, was also frequently detected [15]. The pathogenic bacteria were *Staphylococcus aureus*, *Enterobacter cloacae*, *Enterococcus* spp. In 2 cases (33.3%) with intra-abdominal abscess in the supra-pancreatic area after distal gastrectomy with D2 lymph-node dissection, the same bacteria (*α-Streptococcu*s spp. and *Haemophilus parainfulenzae*) were detected in the drainage fluid and periodontal pocket. From the above results, the null hypothesis was rejected. These abscess formations may have been based on pancreatic fistulae. Other causative bacteria of intra-abdominal infection included *Staphylococcus aureus*, *Enterococcus sp*., *Neisseria spcies*, *Staphylococcus epidermidis*, *Enterobacter cloacae* and *Streptococcus angionosus*.

Table 2 shows the relationship between the severity of PD and intra-abdominal infection in patients with positive stomach cultures. Stomach mucosa cultures were positive in 26 cases; in 20 of these cases, the strain detected in the stomach mucosa was the same as that detected in the periodontal pocket (76.9%).

**Table 2:** The relationship between severity of periodontal disease and intra-abdominal infection after surgery.

Periodontal Disease	n	Positive of Stomach mucosa (%)	Same as Oral (%)	≥Grade 2 Intra-abdominal infection (%)
Mild	5	0 (0.0)	0 (0.0)	0 (0.0)
Intermediate	22	11 (50.0)	9 (81.8)	3 (13.6)
Severe	25	15 (60.0)	11 (73.3)	4 (16.0)

## Discussion

Our results revealed three important findings. Firstly, the bacterial environment in the periodontal pocket and stomach mucosa are similar; the same bacterial strains were detected in 76.9% of cases. Secondly, stomach mucosa cultures and intra-abdominal infection were negative in all patients with mild PD. Finally, the bacteria in the periodontal pocket can directly cause intra-abdominal infection after gastrectomy.

Swallowing of bacteria in the periodontal pocket may be a reason for bacterial coincidence between the oral cavity and stomach mucosa. Indeed, previous papers have reported that more than 10^8^/g bacteria existed in the dental plaque and that 8.3×10^6^/mL of *Porphyromonas gingivalis* was present in the saliva of patients with PD [16]. The total area of the periodontal pocket increases with the progression of PD. For example, a periodontal pocket of 5 mm in 28 teeth could make wide space of approximately 72 mm^2^, for dental plaque to accumulate [17]. It is hypothesized that patients with severe PD would swallow a much larger amount of bacteria than those with mild PD. In fact, bacteria were not detected in the stomach mucosa of patients with mild PD in our study. Oral management focusing on treating PD and reducing the amount of bacteria in the periodontal pocket before surgery might prevent intra-abdominal infection after gastrectomy. Generally speaking, the intra-stomach environment is too strong acid for bacteria to exist [18, 19]. With the exception of *Helicobacter pylori* with urease activity, it is difficult for most bacteria to grow in this environment [19–21]. However, in the present study, bacterial cultures from the stomach mucosa were positive in 26 cases (50%) despite the administration of prophylactic antibacterial drugs before surgery. This might be due to decreased gastric acid secretion in patients with gastric cancer [22–26] who often have atrophic gastritis or take proton pomp inhibitors before surgery [25–28].

In addition, anaerobic bacteria or obligatory anaerobes tend to grow in the deeper periodontal pocket and are unlikely to be presence on the surface of the tooth or tongue. Anaerobic bacteria might have a highly possibility of increasing in number in the intra-abdominal space after gastrectomy. Surgeons should be careful when manipulating the resected stomach and performing anastomosis, because the surgical field might be exposed to bacteria in fluid leaking from the remnant stomach in patients with severe PD. Lavage of the surgical field is also required after anastomosis.

Zilberstein et al. previously reported the results of bacterial culturing of the oral cavity, periodontal pocket and gastric juice [29], and the bacterial flora of dental plaque and gastric juice has been analyzed using a genetic sequencer [30, 31]. However, the bacteriological association between the periodontal pocket and stomach mucosa has been unclear. The present study is the first and only report to show such an association. In order to avoid contamination, we collected strains from the periodontal pocket and gastric mucosa at the same time under sterilize conditions.

The present study was associated with several limitations. First, the concordance of bacteria in the drain fluid and periodontal pocket was observed in only 2 (33.3%) of 6 patients with a positive culture of drain fluid. Other infectious routes of intra-abdominal infection aside from direct exposure to oral bacteria were unclear, as we did not culture bacteria from other sites in this study. Detected bacteria from drainage fluid were mainly streptococcus or staphylococcus strains, it might be bacterial translocation from incisional wound or other part of oral. Second, the culture method was insufficient. Some bacteria require a specific medium or environment, and 2−4 weeks are required, which is longer in comparison to usual methods [32]. Genetic analyses might be required to verify that the same strains were present both in the periodontal pocket and stomach. In addition, only two patients showed the same bacteria in the drain fluid and periodontal pocket. The incidence of events was low, making it difficult to consider other potential confounding factors [7, 33, 34].

## Conclusion

We found the association that the same bacterial strains were detected in the periodontal pocket, the stomach mucosa and drain fluid. Careful preoperative oral management might be particularly important in patients with severe PD.

## Supporting information

S1 File (CSV)
